# Quantum-enhanced detection of viral cDNA via luminescence resonance energy transfer using upconversion and gold nanoparticles

**DOI:** 10.1515/nanoph-2024-0663

**Published:** 2025-03-31

**Authors:** Shahriar Esmaeili, Navid Rajil, Ayla Hazrathosseini, Benjamin W. Neuman, Masfer H. Alkahtani, Dipankar Sen, Qiang Hu, Hung-Jen Wu, Zhenhuan Yi, Robert W. Brick, Alexei V. Sokolov, Philip R. Hemmer, Marlan O. Scully

**Affiliations:** Institute for Quantum Science and Engineering, Texas A&M University, College Station, TX 77843, USA; Department of Physics and Astronomy, Texas A&M University, College Station, TX 77843, USA; Department of Biology, Texas A&M University, College Station, TX 77843, USA; King Abdulaziz City for Science and Technology (KACST), Riyadh 11442, Saudi Arabia; Department of Chemical Engineering, Texas A&M University, College Station, TX 77843, USA; Department of Electrical and Computer Engineering, Texas A&M University, College Station, TX 77843, USA

**Keywords:** luminescence resonance energy transfer (LRET), upconversion nanoparticles, SARS-CoV-2, cDNA, quantum sensing

## Abstract

The COVID-19 pandemic has profoundly impacted global economies and healthcare systems, revealing critical vulnerabilities in both. In response, our study introduces a sensitive and highly specific detection method for cDNA, leveraging Luminescence Resonance Energy Transfer (LRET) between upconversion nanoparticles (UCNPs) and gold nanoparticles (AuNPs), and achieves a detection limit of 242 fM for SARS-CoV-2 cDNA. This innovative sensing platform utilizes UCNPs conjugated with one primer and AuNPs with another, targeting the 5′ and 3′ ends of the SARS-CoV-2 cDNA, respectively, enabling precise differentiation of mismatched cDNA sequences and significantly improving detection specificity. Through rigorous experimental analysis, we established a quenching efficiency range from 10.4 % to 73.6 %, with an optimal midpoint of 42 %, thereby demonstrating the superior sensitivity of our method. Our work uses SARS-CoV-2 cDNA as a model system to demonstrate the potential of our LRET-based detection method. This proof-of-concept study highlights the adaptability of our platform for future diagnostic applications. Instrumental validation confirms the synthesis and formation of AuNPs, addressing the need for experimental verification of the preparation of nanomaterial. Our comparative analysis with existing SARS-CoV-2 detection methods revealed that our approach provides a low detection limit and high specificity for target cDNA sequences, underscoring its potential for targeted COVID-19 diagnostics. This study demonstrates the superior sensitivity and adaptability of using UCNPs and AuNPs for cDNA detection, offering significant advances in rapid, accessible diagnostic technologies. Our method, characterized by its low detection limit and high precision, represents a critical step forward in developing next-generation biosensors for managing current and future viral outbreaks. By adjusting primer sequences, this platform can be tailored to detect other pathogens, contributing to the enhancement of global healthcare responsiveness and infectious disease control.

## 1 Introduction

The COVID-19 pandemic has presented profound challenges to global health and economic stability. Since its emergence in December 2019, the pandemic has resulted in over 135 million confirmed cases and 2.9 million deaths globally as of April 2021 [1]. Effective testing remains essential for managing patient care, controlling the pandemic, and implementing measures to limit viral spread, such as identifying and isolating infected individuals, contact tracing, and surveillance. However, current methods for SARS-CoV-2 detection face several challenges and limitations [2], [3].

RT-PCR is widely regarded as the gold standard for SARS-CoV-2 testing due to its high sensitivity and specificity [4]. Despite its advantages, RT-PCR testing has limitations, including the need for specialized equipment and skilled personnel, long processing times, and susceptibility to false negatives [5], [6], [7].

Nanotechnology-based biosensors offer promising alternatives to traditional diagnostic methods, providing rapid, cost-effective, and user-friendly options for detecting SARS-CoV-2. Techniques such as lateral flow assays, surface-enhanced Raman scattering, luminescence resonance energy transfer (LRET), and electrochemical biosensors have shown potential in overcoming some of the limitations of existing diagnostic tools. For example, Song et al. [8] developed an LRET-based biosensor for the multiplexed detection of SARS-CoV-2 RNA, achieving detection limits of 15 pM and 914 pM for ORF and N genes, respectively. This method enhances detection efficiency and reduces false negatives by analyzing two gene fragments simultaneously, offering significant advantages in COVID-19 diagnostics [9], [10], [11], [12], [13].

Luminescence resonance energy transfer (LRET) is a sensitive, distance-dependent technique widely applied in biomedical and clinical research. LRET involves nonradiative energy transfer from a donor fluorophore to an acceptor chromophore, typically over distances of 1–10 nm. This distance sensitivity makes LRET highly suitable for studying molecular interactions and proximity-based assays [14], [15], [16], [17], [18]. LRET-based biosensors have been employed in detecting specific nucleic acid sequences, with the nucleic acid targets bridging the donor and acceptor particles upon hybridization, thereby generating a measurable LRET signal [19], [20], [21], [22], [23]. cDNA detection using LRET has applications across various domains, including gene expression analysis, SNP detection, and pathogen identification [24], [25].

In this study, we present a novel LRET-based detection method utilizing upconversion nanoparticles (UCNPs) and gold nanoparticles (AuNPs) for the precise detection of SARS-CoV-2 cDNA. We use SARS-CoV-2 cDNA as a model system to demonstrate the potential of our platform, which can be adapted for other pathogens by modifying the primer sequences. While this work is a proof of concept, it highlights the versatility of our approach for future diagnostic applications. Our design was inspired by the ultrasensitive detection methods described by Tsang et al. [26], who demonstrated the efficacy of nanoparticle-based biosensing in viral diagnostics. To enhance the performance and stability of our biosensor, we synthesized hydrophilic UCNPs using the 2,2′-[ethylenebis(oxy)] bisacetic acid (EBAA) method. This approach improves the biocompatibility and operational stability of UCNPs, thereby increasing the overall efficacy of the LRET-based assay.

Upconversion nanoparticles (UCNPs) offer unique quantum properties that are advantageous for advanced biosensing applications. These nanoparticles can convert low-energy infrared photons into higher-energy visible or ultraviolet photons through a quantum mechanical process known as upconversion. By altering the composition and structure of UCNPs, we can fine-tune the emission to achieve highly sensitive and specific detection of target molecules [27]. In our study, UCNPs serve as the donor in the LRET mechanism, providing the necessary luminescent properties for effective energy transfer to AuNPs, the acceptor, which enhances the detection sensitivity for viral cDNA.

To validate the LRET mechanism in our system, we provided experimental evidence of efficient energy transfer between UCNPs and AuNPs, which demonstrated robust quenching behavior upon hybridization with target cDNA. Instrumental verification confirmed the successful synthesis of AuNPs, addressing the need for validation in nanomaterial preparation. Furthermore, preliminary tests demonstrated high specificity for SARS-CoV-2 cDNA, with significantly reduced quenching for mismatched sequences, underscoring the selectivity of our approach for the target sequence. This adaptability means that, by modifying the primer sequences, this platform can be tailored to detect other viral or pathogen-specific sequences as needed, making it a versatile tool for future diagnostic applications.

The combination of gold nanoparticles’ optical properties with the minimal autofluorescence and enhanced emission capabilities of UCNPs results in a detection system with exceptional sensitivity and selectivity for SARS-CoV-2 cDNA, achieving a detection limit of 242 fM. Our assay exhibits a clear dose–response relationship, with an optimal midpoint quenching efficiency of 42 % at a cDNA concentration of 36.54 pM. These findings position our LRET-based method as a potentially powerful tool for rapid and accessible diagnostic applications, significantly advancing the field of virus detection and enhancing global preparedness for viral outbreaks.

## 2 Materials and methods

### 2.1 Activation of carboxyl-functionalized UCNPs with EDC/sulfo-NHS

The synthesis of carboxyl-functionalized UCNPs is detailed in the Supplementary Document. To activate COOH-UCNPs using EDC/NHS, 0.5 mg/mL of UCNPs was mixed with an aqueous EDC/NHS solution, following established protocols [17], [28]. Our COOH-UCNPs, synthesized using the EBAA method and composed of LiYF_4_Yb3+(18 %), Er (1.5 %), and Tm (0.5 %) with carboxyl functionalization, have an average diameter of approximately 11 nm. The preparation procedure for these particles is also provided in the Supplementary Information. For activation, 2 mL of COOH-UCNPs was combined with 10 μL of 0.3 mg/μL NHS and 10 μL of 0.2 mg/μL EDC solutions. The solution was vortexed briefly and then agitated vigorously at 500 rpm for 0.5 h using an Eppendorf MixMate or similar shaker. After activation, the particles were centrifuged at 5,080 rcf (9,000 rpm) for 10 min. Approximately 95 % of the supernatant was removed and replaced with fresh DI water. The particles were then resuspended by sonication for about 20 min and evaluated for dispersion using a 980 nm laser, holding the vial in front of the laser beam for visual inspection. Additional details on the experiment can be found in the Supplementary Document.

### 2.2 Conjugation of activated-UCNPs with amino-modified oligonucleotide

The carboxyl-functionalized UCNP (LiYF_4_:Yb3+(18 %), Er (1.5 %), Tm (0.5 %)) was covalently conjugated with an amino-modified oligonucleotide probe. The UCNP-to-oligonucleotide ratio used in this study was 1:10, optimized to ensure efficient binding. The amino-modified oligonucleotide solution, acquired from Integrated cDNA Technologies (IDT), had a molarity of 100 μM. For conjugation, 78 μL of the amino-modified oligonucleotide solution (equivalent to 4.67 × 10^13^ oligonucleotides) was mixed with 1.3 mg of activated UCNPs (equivalent to 4.67 × 10^12^ particles). This mixture was incubated overnight at 4 °C under constant agitation to facilitate binding. Following incubation, the particles were washed with DI water three times using centrifugation at 5,080 rcf (9,000 rpm) for 10 min each. More details on the conjugation process are provided in the Supplementary Document.

### 2.3 Conjugation of thiol-modified primer with AuNPs

This protocol describes the labeling of 5 nm gold nanoparticles (AuNPs) with thiolated oligonucleotides. A 1:10 ratio of AuNPs to thiol-modified oligonucleotides was maintained, with a mass concentration of 5 nm AuNPs around 0.06 mg/mL, corresponding to approximately 4.43 × 10^13^ particles/mL. The thiol-modified oligonucleotide stock solution from IDT had a molarity of 100 μM, and 16 μL of this solution contained approximately 9.6 × 10^14^ oligonucleotides. Prior to conjugation, 30 μL of TCEP was centrifuged at 50 g to remove the supernatant, and the gel was washed twice with DDI water. Next, 16 μL of thiolated oligonucleotide stock solution was added to the TCEP gel, vortexed for 3 min, and incubated for 1 h to ensure complete reduction.

After centrifuging the microtube and recovering the supernatant containing reduced oligonucleotide, 2.17 mL of AuNPs were added to the oligo-TCEP mixture. This mixture was incubated for 2 h to facilitate conjugation [29]. The resultant product was washed three times using an ultracentrifuge (rotor TLA-110) at 110 K RPM for 10 min to remove excess oligos. The final pellet was resuspended in DI water and stored for further analysis [30]. Detailed information on the conjugation process is provided in the Supplementary Document.

### 2.4 Detection of cDNA

The LRET mechanism in our assay involves monitoring fluorescence quenching at key UCNP emission peaks, including 457 nm, 523 nm, 550 nm, 667 nm, and 792 nm. These peaks are chosen based on the spectral overlap between the UCNP fluorescence emission and the absorption spectrum of AuNPs, which governs the efficiency of energy transfer. Notably, the 550 nm emission peak exhibits the strongest quenching due to optimal spectral overlap, while quenching at 667 nm and 792 nm is weaker. This selection of key emission peaks ensures sensitive detection of target cDNA.

The sensor system used in this study included UCNP-primer 2 and AuNP-primer 1 conjugates, along with the target cDNAs and control sequences, including a 24-base mismatched cDNA (cDNA-mmP1P2) and a 12-base mismatch cDNA (cDNA-mmP1 and cDNA-mmP2). Detection was carried out by mixing UCNP-primer 2 and AuNP-primer 1 conjugates with the target cDNA in DI water at room temperature (25 °C) as described in Sections 2.2 and 2.3. The measured zeta potential values, as detailed in Section 1.3 of the Supplementary File and consistent with those reported by Tsang et al. [26], confirm the stability and functionality of our nanoparticles under these conditions.

The experiment was divided into two parts. In the first part, we evaluated the dose–response of the sensor by maintaining a constant concentration of the sensor while reducing the target cDNA concentration from 5.06 μM to 5.06 fM (as shown in Table 1). In the second part, we assessed the specificity of the sensor for SARS-CoV-2 cDNA by testing three different mismatch controls (cDNA-mmP1P2, cDNA-mmP1, and cDNA-mmP2) at a concentration of 5.06 μM.

**Table 1:** Quantities of experimental materials used in the LRET-based SARS-CoV-2 cDNA detection study. The final concentration of cDNA is varied while the number of upconversion nanoparticles (UCNPs), UCNP-conjugated primers, gold nanoparticles (AuNPs), and AuNP-conjugated primers remain constant at 4.7 × 10^12^, 4.7 × 10^13^, 2.35 × 10^13^, and 2.35 × 10^14^, respectively.

Final concentration of cDNA [M]	Number of target cDNA
Cntl: 0	0
5.06 × 10^−6^	3.6 × 10^14^
5.06 × 10^−8^	3.6 × 10^12^
5.06 × 10^−11^	3.6 × 10^9^
5.06 × 10^−12^	3.6 × 10^8^
5.06 × 10^−14^	3.6 × 10^6^
5.06 × 10^−15^	3.6 × 10^5^

The experimental procedure began by combining the target cDNAs with amino-modified UCNPs and incubating the mixture at room temperature for 2 h. Following this incubation, the solution was combined with a thiol-modified AuNP solution at a 1:5 particle ratio and mixed thoroughly for 15 min. Luminescence spectra were recorded using continuous-wave stimulation at 980 nm. Table 1 provides detailed information on the materials used in the experiment, including the concentrations and quantities of UCNPs, UCNP-conjugated primers, target cDNA sequences, AuNPs, and AuNP-conjugated oligonucleotides.

The concentrations of cDNA used in the study were selected to cover a wide range of values, spanning several orders of magnitude (as shown in Table 1). This design ensures that the dose–response of the assay can be accurately assessed, allowing us to evaluate the system’s sensitivity and detection limits. The specific concentrations were determined based on the optimal ratio of UCNP:DNA:AuNP, which ensures that the quenching efficiency is measurable and reflective of the target interactions. The chosen concentrations are essential for understanding the limits of detection and providing a comprehensive characterization of the assay’s performance.

## 3 Results and discussion

**Figure 1:**
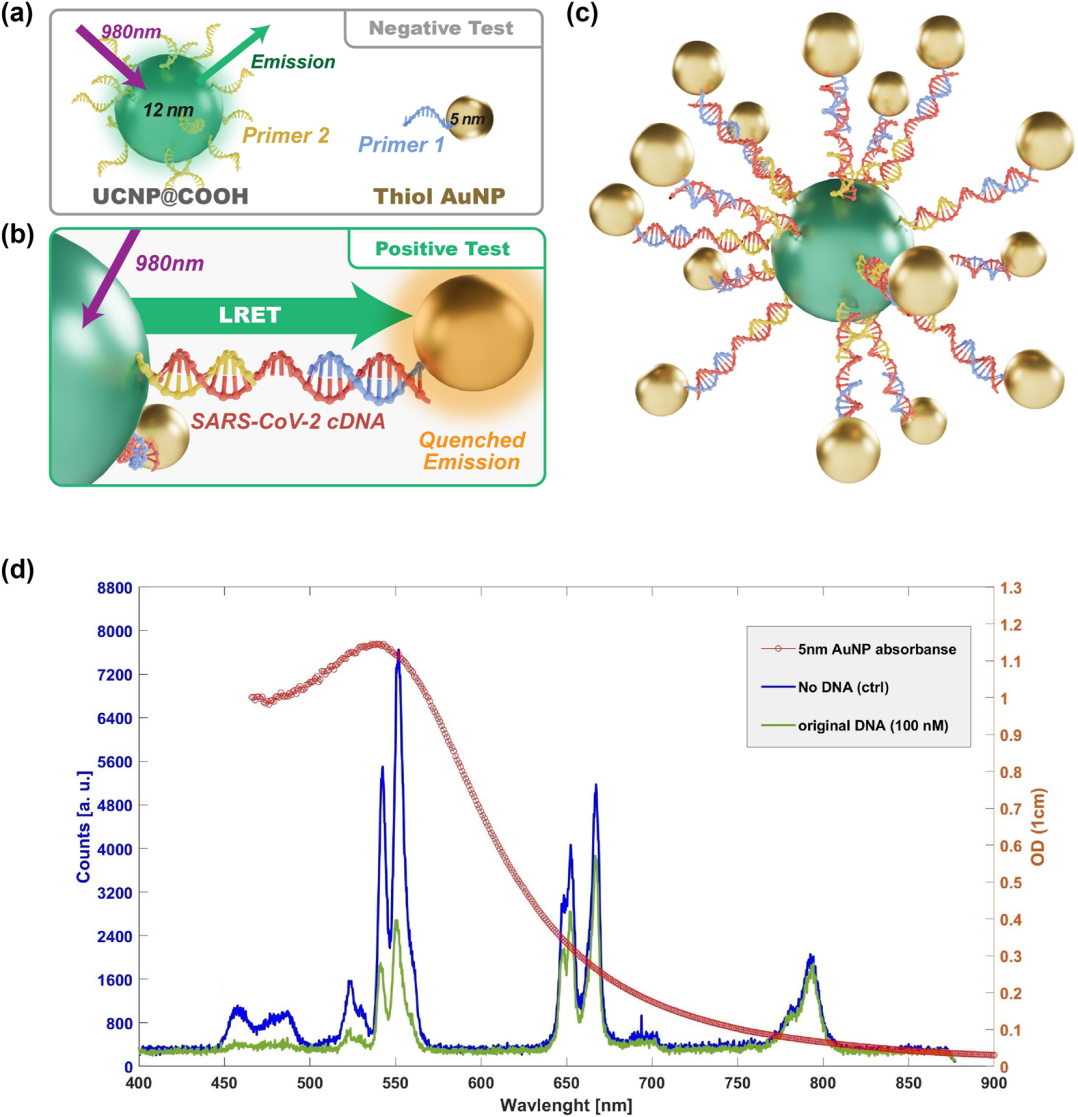
Feasibility testing of the assay: (a) schematic representation of the control test where the cDNA is absent, preventing UCNP-AuNP binding, thereby resulting in higher fluorescence intensity from the UCNPs. (b) Schematic of the positive test with cDNA present, facilitating UCNP-AuNP binding and resulting in reduced fluorescence intensity from UCNPs due to LRET-induced quenching. (c) 3D schematic of conjugated UCNPs surrounded by conjugated AuNPs linked by SARS-CoV-2 cDNA. (d) Overlay of the AuNP absorption spectrum and UCNP fluorescence spectrum for both control (no cDNA) and positive tests (with cDNA, 100 nM). The positive test demonstrates lower fluorescence, especially around the 550 nm wavelength, while quenching effects are less prominent at 667 nm and 792 nm wavelengths.

To validate the functionality and feasibility of our LRET-based assay, we initially conducted both positive and negative (control) tests. These preliminary tests ensured that the particles and the target SARS-CoV-2 cDNA were binding correctly, providing a foundation for accurate dose–response measurements. For the negative (control) test, we prepared a sample consisting of UCNPs conjugated with primer 2 and AuNPs conjugated with primer 1, and we added 6 μL of DI water to this mixture (Figure 1). This setup served as a baseline, representing conditions without target cDNA.

For the positive test, we utilized the same concentration and volume of UCNPs and AuNPs as in the control but added 6 µL of SARS-CoV-2 cDNA at a stock solution concentration of 10^−4^ M, resulting in a final cDNA concentration of 5.06 μM (Table 1 and Figure 1). As mentioned, the key emission peaks (550 nm, 457 nm, and 523 nm) were monitored to evaluate LRET-induced quenching, with the 550 nm peak showing the most significant quenching due to optimal spectral overlap between UCNP emission and AuNP absorption. This setup simulated conditions where the target cDNA sequence is present, allowing us to observe LRET-based fluorescence quenching as an indicator of cDNA binding.

In the absence of target cDNA, the AuNPs did not bind to the UCNPs through cDNA-primer hybridization, which allowed for a higher fluorescence intensity from the UCNPs (Figure 1). Conversely, in the presence of the specific cDNA sequence, cross-linking between the UCNPs and AuNPs occurred, facilitating LRET coupling. This coupling led to quenching of the fluorescence signal from the UCNPs, as illustrated in the positive test (Figure 1), with a notably lower fluorescent intensity observed around the 550 nm wavelength.

Our analysis focused on key emission peaks at 457 nm, 523 nm, 550 nm, 667 nm, and 792 nm. This selection enabled us to probe various regions within the absorption spectrum of AuNPs and assess their involvement in LRET coupling. As shown in Figure 1(d), the absorption cross section of AuNPs is superimposed with the fluorescence spectrum of UCNPs. Notably, the quenching observed at 550 nm was more pronounced than at 667 nm and 792 nm, consistent with the absorption characteristics of the AuNPs. The highest quenching occurred at 550 nm, where the overlap between the emission of UCNPs and absorption of AuNPs is maximal.

The reduced quenching efficiency observed at 667 nm and 792 nm can be attributed to the spectral properties of the 5 nm AuNPs. As shown, the maximum absorption for 5 nm AuNPs is centered around 520 nm, which aligns closely with the 550 nm emission peak of UCNPs. In contrast, the absorption cross sections for 667 nm and 792 nm are considerably lower, resulting in reduced LRET-induced quenching at these wavelengths [31]. This behavior underscores the importance of the spectral overlap between donor and acceptor particles in determining LRET efficiency, validating the design of our assay in targeting specific wavelengths for optimal sensitivity.

These preliminary tests confirm the feasibility of our LRET-based assay, with a clear distinction between the fluorescence intensities of positive and control samples. This initial validation provided a solid foundation for subsequent dose–response experiments, demonstrating that our assay could differentiate between the presence and absence of target cDNA based on LRET-induced fluorescence quenching. Our findings, particularly the robust quenching observed at 550 nm, highlight the potential of this assay for sensitive and specific detection of SARS-CoV-2 cDNA, making it a promising tool for viral diagnostics.

**Figure 2:**
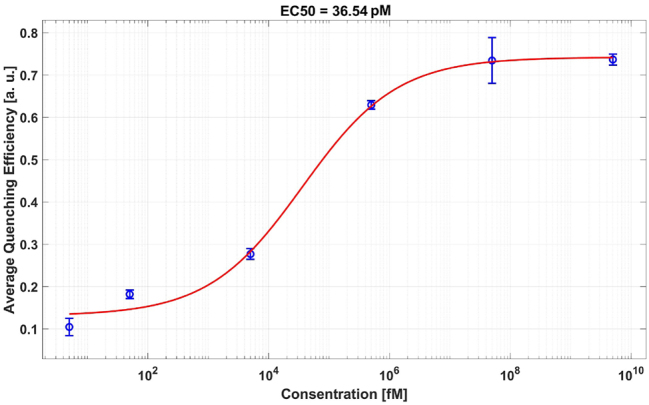
Dose–response curve depicting quenching efficiency for each tested concentration, as detailed in Table 1. The curve indicates a well-defined dynamic range and sensitivity of the assay, with a detection limit of 242 fM and an EC50 value of 36.54 pM. Error bars represent the standard deviation (SD) from three independent replicates (*n* = 3).

To evaluate the dynamic range and sensitivity of our LRET-based assay, we mixed UCNPs and AuNPs with SARS-CoV-2 cDNA at varying concentrations as outlined in Table 1. The dose–response curve, shown in Figure 2, captures the quenching efficiency across a range of cDNA concentrations, from 5.06 fM to 5.06 μM. We defined the quenching efficiency at 5.06 fM, plus three times the standard deviation of the same data point, as the assay’s detection limit. Through a four-parameter logistic regression fit to the dose–response data, we determined the limit of detection to be 242 fM, with an EC50 (midpoint quenching efficiency) value of 36.54 pM.

The red curve in Figure 2 represents the four-parameter logistic fit, commonly used for modeling sigmoidal dose–response curves in biosensing applications due to its ability to capture nonlinear binding dynamics, including cooperative and noncooperative interactions [32].

(1) $$Y=\text{Min}+\frac{\text{Max }-\text{ Min}}{1+{\left(\frac{x}{\text{EC}50}\right)}^{hc}}$$

Here, Min and Max denote the minimum and maximum quenching efficiencies, *x* is the cDNA concentration, EC50 is the concentration at which the quenching efficiency reaches $\frac{\text{Max }+\text{ Min}}{2}$ , and *hc* is the Hill coefficient. The 4PL fit is appropriate for describing the sigmoidal quenching observed in our system, as it accounts for variable binding behaviors. The resulting EC50 from the fit was 36.54 pM, with a Hill coefficient of −0.5517. Quenching efficiency (QE) for each concentration was calculated as:

(2) $$\text{QE}=\frac{I_{\text{neg}}-I_{\text{conc}}}{I_{\text{neg}}}$$

where *I*_neg_ is the fluorescence intensity at 550 nm in the negative control, and *I*_conc_ is the intensity at 550 nm for the tested cDNA concentration.

To contextualize the sensitivity of our assay, we analyzed the number of UCNPs and AuNPs required to detect a single cDNA molecule in a positive sample. This analysis hinges on the detection limit established in Figure 2, where quenching efficiency ranges from 10.4 % to 73.6 %, with a midpoint of 42 % corresponding to a cDNA concentration of 36.54 pM. Using this concentration in a reaction volume of 119 μL, we calculated the equivalent of 2.6 × 10^9^ cDNA molecules (see Supplementary Materials). Given the fixed UCNP and AuNP quantities across cDNA concentrations, the optimal cDNA:UCNP:AuNP ratio for extrapolating the detection of a single cDNA molecule was determined to be approximately 1:1.8 × 10^3^:9 × 10^3^. This implies that, under our experimental conditions, detecting a single cDNA molecule would theoretically require 1.8 × 10^3^ UCNPs and 9.0 × 10^3^ AuNPs. We maintained a consistent UCNP-to-AuNP ratio of 1:5 throughout the experiment.

To investigate the specificity of our assay, we prepared various mismatched cDNA sequences. Our assay design includes primers that bind specifically to the 5′ and 3′ ends of the SARS-CoV-2 cDNA. We created three types of mismatched cDNA: (1) cDNA-mmP1P2, with mismatches on both ends (24 mismatched bases out of 42); (2) cDNA-mmP2, with mismatches only on the UCNP-bound side (12 mismatched bases out of 42); and (3) cDNA-mmP1, with mismatches only on the AuNP-bound side (12 mismatched bases out of 42) (see Supplementary Materials for sequences). The number of mismatches was chosen to represent two levels of specificity testing: minimal mismatches (12 bases) to assess sensitivity to small mutations and extensive mismatches (24 bases) to simulate highly nonspecific binding. This design ensures that the assay can detect target cDNA while minimizing false positives.

**Figure 3:**
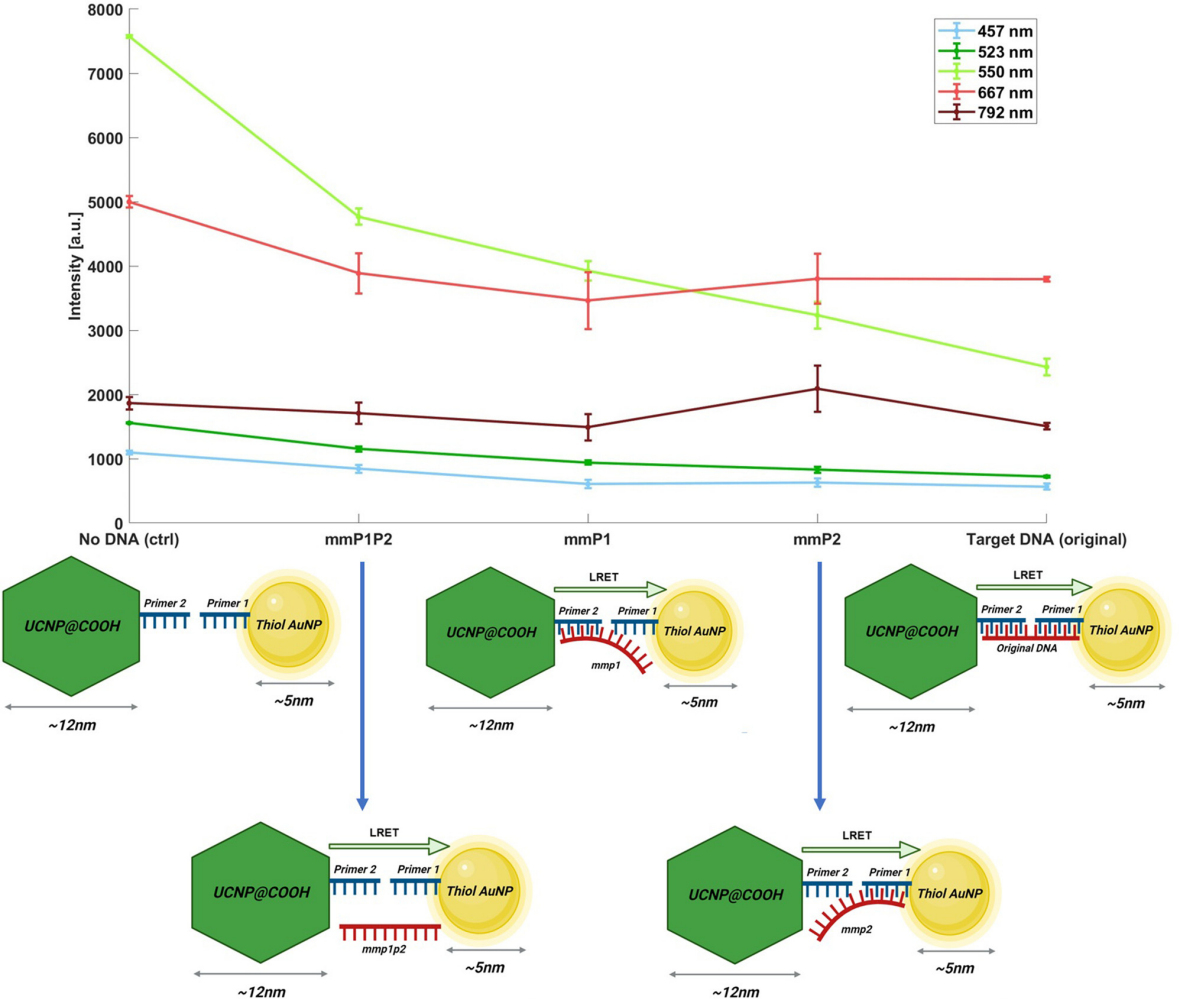
Specificity analysis of the assay using different mismatched cDNA sequences. Average intensity at major UCNP peaks for each mismatched cDNA sequence type. The target cDNA shows the highest quenching (64.4 %) relative to the negative control, while the mmP1P2 sequence (mismatch on both sides) shows only 39.3 % quenching. The results are based on three replicates, and error bars indicate standard deviations.

When both ends were mismatched (cDNA-mmP1P2), quenching at 550 nm resulted in a reduction of 2,984 counts (39.3 % quenching efficiency). In contrast, the fully complementary target cDNA caused a more substantial reduction of approximately 4,907 counts (64.6 % quenching efficiency), relative to the negative control (Figure 3). For cDNA-mmP1, where only the UCNP-binding region was mismatched, the quenching efficiency at 550 nm was 50.1 %, with an observed intensity reduction of 3,806 counts relative to the control. For cDNA-mmP2, where only the AuNP-binding region was mismatched, the quenching efficiency was 53.9 %, with an observed reduction of 4,093 counts.

These variations in quenching efficiency can be attributed to differences in binding affinity between each mismatched cDNA sequence and the primers. Higher cDNA concentrations can yield significant binding ratios despite lower affinities. Additionally, our results underscore the high specificity of our assay, as demonstrated by the higher quenching for the fully complementary target cDNA compared to mismatched sequences.

Further studies are warranted to elucidate the precise relationship between quenching efficiency, sequence mismatches, and mismatch location. Notably, quenching behavior at 457 nm and 523 nm wavelengths mirrored that of 550 nm, while no substantial quenching was observed at 792 nm or 667 nm for any sample. The observed quenching trends suggest that the target cDNA exhibits the highest affinity, followed by single-mismatch sequences (mmP1 and mmP2) with intermediate affinities, and the lowest affinity is observed for the double-mismatch sequence (mmP1P2). These findings support the expected correlation between sequence complementarity and binding strength while highlighting the need for future experimental validation to determine absolute binding affinities.

These findings demonstrate the feasibility and sensitivity of our LRET-based detection method highlighting its potential for precise SARS-CoV-2 cDNA detection. Future studies will focus on optimizing experimental conditions such as pH, reagent concentrations, temperature, nanoparticle and primer loading, and PEG coating density, along with cross-detection studies and performance evaluations using real-world samples.

An essential finding from our specificity test is that the location of mismatched bases significantly impacts the binding efficiency and quenching response of the assay. This is evident when comparing cDNA-mmP2 (mismatched on the UCNP side only) with cDNA-mmP1P2 (mismatched on both the UCNP and AuNP sides) and cDNA-mmP1 (mismatched on the AuNP side only). Results indicate that mismatches on the UCNP side alone have a lesser impact on hybridization compared to the other two cases, suggesting that the AuNP-side primer is more critical in influencing binding specificity.

To interpret the results of the mismatch experiments, it is essential to understand that quenching in this assay is a result of the binding between gold nanoparticles (AuNPs) and upconversion nanoparticles (UCNPs), which is mediated by the affinity of the cDNA to be detected. The concentration of cDNA relative to nanoparticle-bound primers, which remains constant, plays a key role. We applied the Langmuir equation to qualitatively describe cDNA binding to the primers:

(3) $$\theta =\frac{\left[\text{cDNA.Primer}\right]}{{\left[\text{Primer}\right]}_{\text{total}}}\approx \frac{{\left[\text{cDNA}\right]}_{\text{total}}}{K_{d}+{\left[\text{cDNA}\right]}_{\text{total}}}$$

where [cDNA.Primer] denotes the concentration of cDNA bound to both primers, [Primer]_total_ represents the total concentration of primers (either UCNPs or AuNPs, adjusted for units), and [cDNA]_total_ refers to the total cDNA concentration (target, mmP1, mmP2, or mmP1P2). This equation helps provide an intuitive framework for predicting quenching efficiency among different cDNA sequences, as stronger binding affinity is expected to correlate with lower dissociation constants (*K*_*d*_).

The logical basis for binding affinities follows standard DNA hybridization physics. The affinity of the fully complementary target cDNA to both primers is the highest due to specific hydrogen bonding interactions, leading to maximum quenching. Sequences with a single mismatch (either in primer 1 or primer 2) have reduced affinity, resulting in moderate quenching. The lowest affinity is observed in the case of double mismatches (mmP1P2), where both binding sites are disrupted, leading to minimal quenching close to the control condition with no cDNA present.

The binding affinity trend follows logical expectations based on DNA sequence complementarity. The strongest binding occurs when the target cDNA is fully complementary to both primers, leading to the highest quenching. As mismatches are introduced – either in primer 1 or primer 2 – the binding affinity decreases, resulting in reduced quenching. The lowest affinity is observed when both primer binding sites contain mismatches (mmP1P2), leading to minimal quenching close to the control condition with no cDNA present. While theoretical calculations help establish this expected trend, precise experimental determination of *K*_*d*_ values is necessary for quantitative validation. Additionally, optimizing the concentration of cDNA and primers in each case will further enhance the robustness of the assay.

Although theoretical estimates suggest a clear trend in affinity, accurate experimental determination of *K*_*d*_ values remains essential for quantitative validation. Additionally, optimizing cDNA concentrations for each case could further improve the robustness of the assay, which will be explored in future work.

This suggests that the quenching efficiency, from lowest to highest, should follow cDNA-mmP1P2, cDNA-mmP2, cDNA-mmP1, and target cDNA. However, it is critical to recognize that theoretical models inherently assume idealized conditions and may overestimate binding affinities. Despite this, we observe that even the sequence with the lowest predicted affinity (cDNA-mmP1P2) retains significant binding activity, reflected by a measurable quenching efficiency.

In this experiment, the presence of UCNP-primer 2-mismatch cDNA-primer 1-AuNP complexes is detected by spectral measurements, comparing results to the negative control using Equation (2). Quenching efficiency is influenced by the concentration of mismatched cDNA and its affinities to both primers. In our setup (Figure 3), we maintained cDNA concentration constant at 5.06 μM to focus on affinity-driven quenching variations. Although one might expect cDNA-mmP1P2 (with mismatches on both sides) to exhibit negligible quenching, our data show otherwise, supporting the notion that even low-affinity mismatches can yield quenching at sufficient concentrations.

The Langmuir equation offers a theoretical basis for this observation:

(4) $$\theta =\frac{\left[\text{cDNA.Primer}\right]}{\left[\text{Primer}\right]}\approx \frac{{\left[\text{cDNA}\right]}_{\text{total}}}{K_{d}+{\left[\text{cDNA}\right]}_{\text{total}}}$$

**Figure 4:**
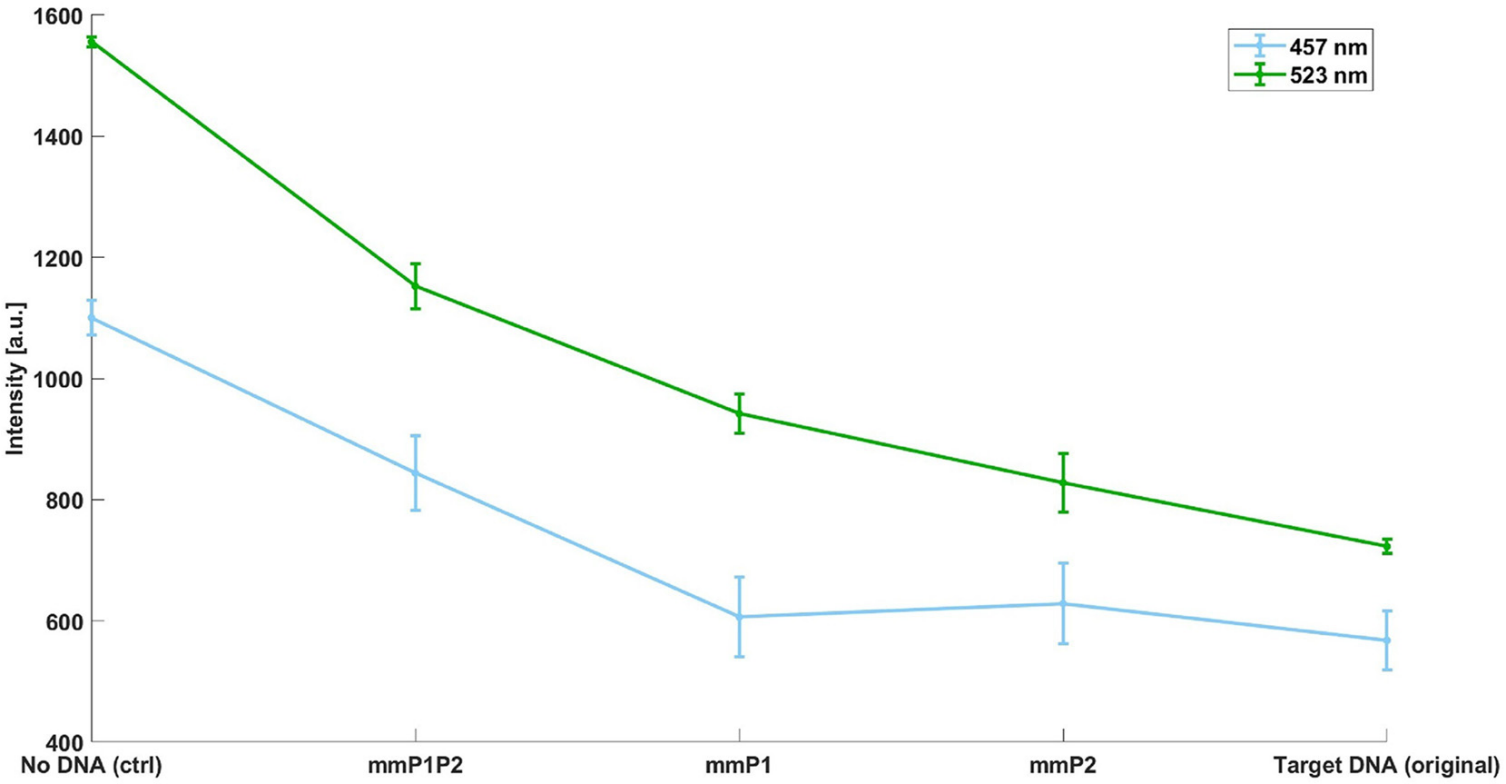
Zoomed-in view of the intensity for 523 nm and 457 nm, showing lower sensitivity compared to 550 nm. The results are based on three replicates, and error bars indicate standard deviations. We used a line format instead of a scatter plot as it better highlights changes in quenching efficiency, especially for wavelengths with lower quenching (523 nm and 457 nm).

With a substantial difference between theoretical estimations of *K*_*d*_ ≈ 10^−16^ M and the concentration 5.06 × 10^−6^ M, *θ* approaches 1, indicating significant complex formation despite theoretical limitations. This helps explain the quenching seen with cDNA-mmP1P2, albeit to a lesser degree than target cDNA (Figure 4).

To ensure clarity, we emphasize that optimizing experimental conditions, particularly cDNA concentrations, is a necessary step for refining this detection method. Future work will focus on validating these binding trends through experimental determination of *K*_*d*_ values and refining concentration-dependent effects under different mismatch conditions.

**Figure 5:**
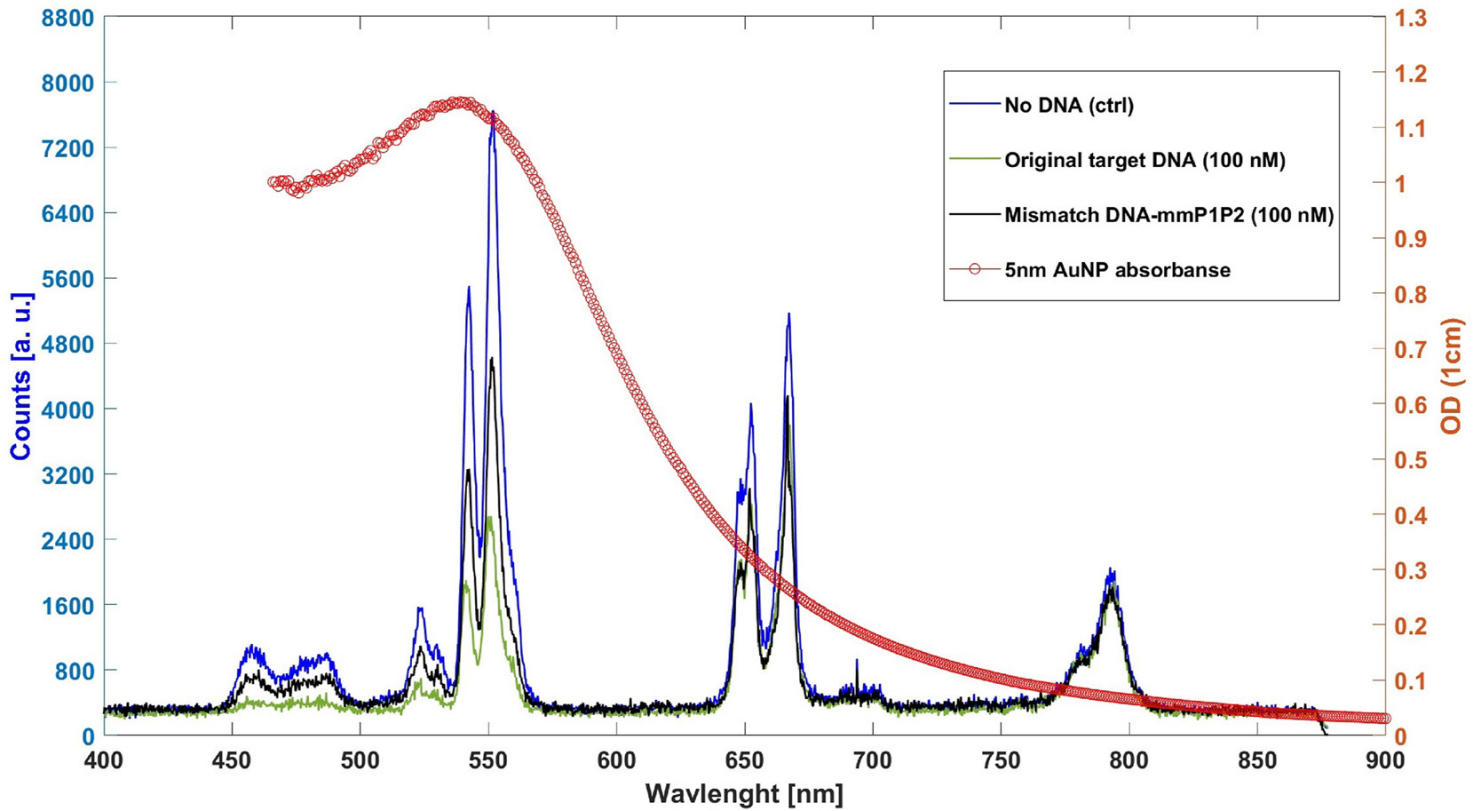
Spectral comparison of UCNP emission for the negative control (no DNA), original target cDNA (100 nM), and mismatch cDNA (cDNA-mmP1P2, 100 nM) samples. The spectra show stronger fluorescence quenching in the positive test compared to the mismatch DNA. The 5 nm AuNP absorbance spectrum is included for reference.

The specificity test reveals that mismatches on both hybridization sites (cDNA-mmP1P2) significantly reduce quenching efficiency by approximately 40 % compared to the fully complementary target cDNA (see Figure 5). Single-side mutations (e.g., cDNA-mmP1) yield a moderate reduction in efficiency, particularly when mismatches occur on the UCNP-binding side, suggesting a minor impact on the efficacy of the test.

**Figure 6:**
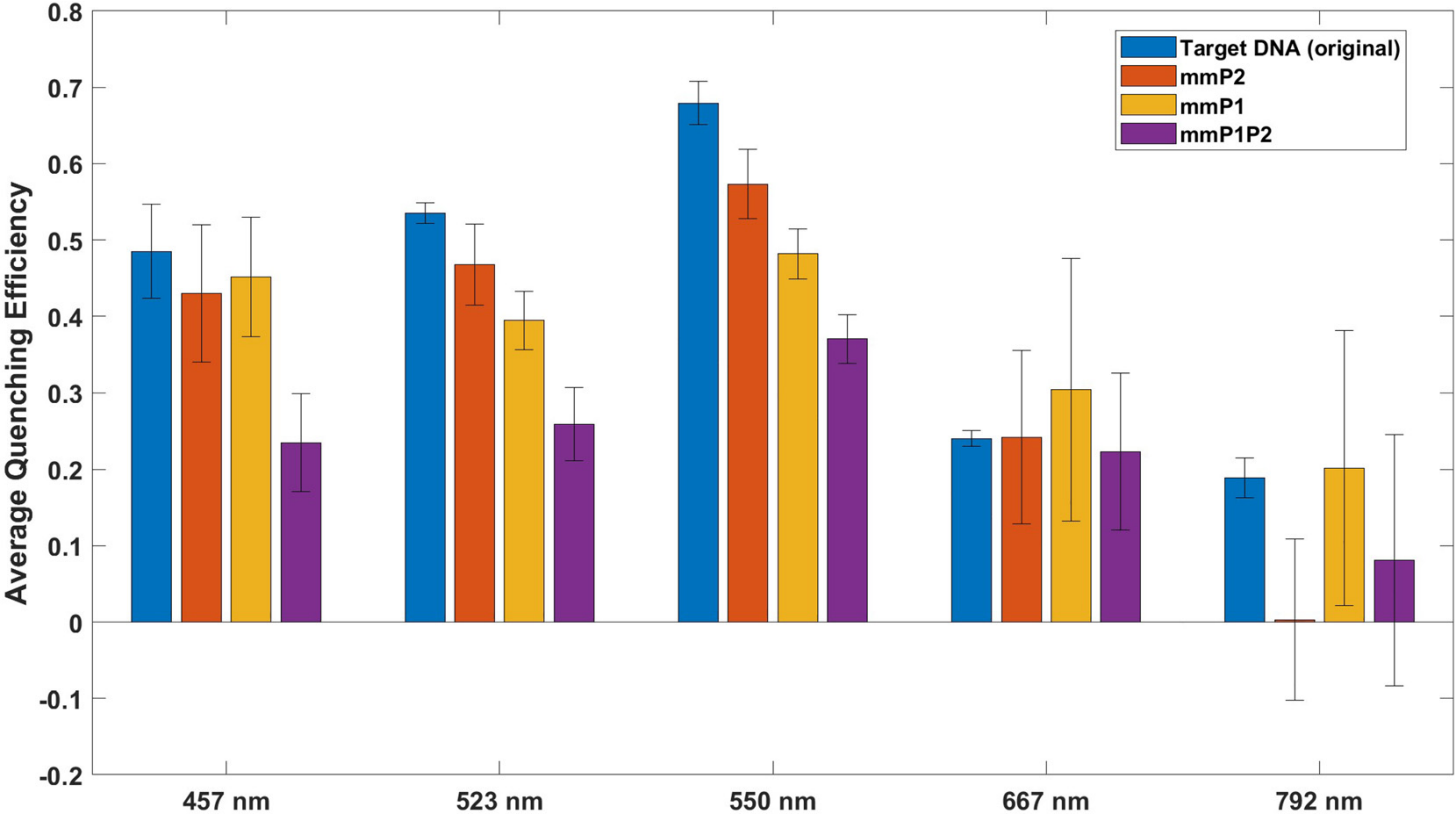
Comparison of quenching efficiencies across various wavelength bands for target and mismatched cDNA sequences (mmP1, mmP2, mmP1P2). Error bars represent standard deviations across three replicates. Although mismatch sequences exhibit some quenching, the fully complementary target sequence displays the highest quenching efficiency across key wavelengths (457 nm, 523 nm, and 550 nm), supporting specificity within the assay’s operational dynamic range. Minimal quenching at 792 nm is consistent with AuNP absorbance characteristics.

Quenching efficiencies across various wavelength bands for both target and mismatched cDNA were examined to confirm the specificity and mechanism of LRET in our assay. As shown in Figure 6, the 792 nm wavelength displayed minimal quenching, aligning with the lower absorbance of AuNPs in this region. In contrast, quenching was most pronounced in the green and blue bands, where AuNP absorbance is highest, further substantiating LRET occurrence in this setup.

Although 980 nm continuous-wave (CW) lasers are known to cause localized heating in aqueous environments, which could potentially interfere with biological processes such as DNA-primer hybridization, we did not observe any instability in our dose–response curves or irregularities in measurement. This indicates that within the laser power and exposure time used in our experiments, heating effects were minimal and did not disrupt the performance of the assay. Nonetheless, we acknowledge the importance of monitoring heating effects and will explore the conditions under which they might impact the assay’s performance. Future work will also consider optimizing the excitation wavelength, such as using an 808 nm laser, to further reduce heating-related risks.

## 4 Conclusions

In this study, we developed and validated a sensitive and specific assay for the detection of SARS-CoV-2 cDNA using a combination of upconversion nanoparticles (UCNPs) and gold nanoparticles (AuNPs). By conjugating UCNPs with one primer and AuNPs with another, we designed a system in which these nanoparticles selectively hybridize with opposite ends of the SARS-CoV-2 cDNA target. This strategic design enables multiple AuNPs to bind to each UCNP upon successful hybridization with the target cDNA, resulting in variable quenching of UCNP fluorescence.

Our assay leveraged the unique optical properties of UCNPs, which emit fluorescence at wavelengths of 426, 524, 551, 666, and 792 nm. The quenching pattern observed, with peak quenching at 551 nm and minimal quenching at 792 nm, aligns with the AuNP absorption spectrum, confirming that the quenching is mediated by luminescence resonance energy transfer (LRET) from UCNPs to AuNPs. This LRET mechanism forms the core of our detection approach, enhancing both the sensitivity and specificity of the assay.

The assay demonstrated a remarkable limit of detection at 242 fM for SARS-CoV-2 cDNA, positioning it as a highly sensitive tool for viral detection. Specificity was rigorously evaluated through tests with mismatched cDNA sequences, showing a significant reduction in quenching efficiency for sequences with mismatches at both the UCNP and AuNP binding sites. This response to mismatched sequences underscores the assay’s robustness and precision in distinguishing the target SARS-CoV-2 cDNA sequence from nonspecific sequences. The midpoint quenching efficiency, measured at 42 % and corresponding to a cDNA concentration of 36.54 pM, serves as a practical threshold for positive detection, further demonstrating the assay’s utility in highly sensitive diagnostic applications.

In summary, our study presents a robust LRET-based assay with a low detection threshold and high specificity, tailored for SARS-CoV-2 detection. While this work is a proof of concept, it highlights the potential of our platform for future diagnostic applications. By modifying the primer sequences, this assay can be adapted to detect other viral nucleic acids, making it a versatile tool for managing current and future viral outbreaks. Future work will focus on expanding this methodology to detect other pathogens and refining the assay for potential clinical and field applications.

## Supplementary Material

Supplementary Material Details

## References

[1] World Health Organization (2023). Coronavirus disease (COVID-19) dashboard. ..

[2] Rajil N. (2022). Quantum optical immunoassay: upconversion nanoparticle-based neutralizing assay for COVID-19. *Sci. Rep.*.

[3] Rajil N. (2021). A fiber optic–nanophotonic approach to the detection of antibodies and viral particles of COVID-19. *Nanophotonics*.

[4] Woloshin S., Patel N., Kesselheim A. S. (2020). False negative tests for SARS-CoV-2 infection – challenges and implications. *N. Engl. J. Med.*.

[5] He X. (2020). Temporal dynamics in viral shedding and transmissibility of COVID-19. *Nat. Med.*.

[6] Lan L. (2020). Positive RT-PCR test results in patients recovered from COVID-19. *Jama*.

[7] Xiao A. T., Tong Y. X., Zhang S. (2020). False negative of RT-PCR and prolonged nucleic acid conversion in COVID-19: rather than recurrence. *J. Med. Virol.*.

[8] Song M. (2022). Multiplexed detection of SARS-CoV-2 based on upconversion luminescence nanoprobe/mxene biosensing platform for COVID-19 point-of-care diagnostics. *Mater. Des.*.

[9] Qiu G., Gai Z., Tao Y., Schmitt J., Kullak-Ublick G. A., Wang J. (2020). Dual-functional plasmonic photothermal biosensors for highly accurate severe acute respiratory syndrome coronavirus 2 detection. *ACS Nano*.

[10] Gupta R. (2020). Nanotechnology-based approaches for the detection of SARS-CoV-2. *Front. Nanotechnol.*.

[11] Seo G. (2020). Rapid detection of COVID-19 causative virus (SARS-CoV-2) in human nasopharyngeal swab specimens using field-effect transistor-based biosensor. *ACS Nano*.

[12] Fabiani L. (2021). Magnetic beads combined with carbon black-based screen-printed electrodes for COVID-19: a reliable and miniaturized electrochemical immunosensor for SARS-Cov-2 detection in saliva. *Biosens. Bioelectron.*.

[13] Moitra P., Alafeef M., Dighe K., Frieman M. B., Pan D. (2020). Selective naked-eye detection of SARS-Cov-2 mediated by N gene targeted antisense oligonucleotide capped plasmonic nanoparticles. *ACS Nano*.

[14] Clegg R. M. (1992). [18] fluorescence resonance energy transfer and nucleic acids. *Methods Enzymol.*.

[15] Roy R., Hohng S., Ha T. (2008). A practical guide to single-molecule FRET. *Nat. Methods*.

[16] Selvin P. R., Ha T. (2008). *Single-Molecule Techniques: A Laboratory Manual*.

[17] Chen Z. (2008). Versatile synthesis strategy for carboxylic acid- functionalized upconverting nanophosphors as biological labels. *J. Am. Chem. Soc.*.

[18] Hemmer P. R. (2023). Opportunities for biosensing with fluorescent diamond and phosphor nanoparticles. *Proc. SPIE PC12447, Quantum Sensing, Imaging, and Precision Metrology*.

[19] Tyagi S., Kramer F. R. (1996). Molecular beacons: probes that fluoresce upon hybridization. *Nat. Biotechnol.*.

[20] Marras S. A. (2006). Selection of fluorophore and quencher pairs for fluorescent nucleic acid hybridization probes. *Fluorescent Energy Transfer Nucleic Acid Probes: Designs and Protocols*.

[21] Tsourkas A., Behlke M. A., Rose S. D., Bao G. (2003). Hybridization kinetics and thermodynamics of molecular beacons. *Nucleic Acids Res.*.

[22] Sperling R. A., Parak W. J. (2010). Surface modification, functionalization and bioconjugation of colloidal inorganic nanoparticles. *Philos. Trans. R. Soc. A Math. Phys. Eng. Sci.*.

[23] Hemmer P. R. (2023). Engineering nanodiamonds for quantum sensing. *Proc. SPIE PC12692, Quantum Communications and Quantum Imaging XXI*.

[24] Hermanson G. T. (2013). *Bioconjugate Techniques*.

[25] Didenko V. V. (2001). DNA probes using fluorescence resonance energy transfer (FRET): designs and applications. *Biotechniques*.

[26] Tsang M.-K., Ye W., Wang G., Li J., Yang M., Hao J. (2016). Ultrasensitive detection of ebola virus oligonucleotide based on upconversion nanoprobe/nanoporous membrane system. *ACS Nano*.

[27] Su Q., Feng W., Yang D., Li F. (2017). Resonance energy transfer in upconversion nanoplatforms for selective biodetection. *Acc. Chem. Res.*.

[28] Dumelin C. E., Scheuermann J., Melkko S., Neri D. (2006). Selection of streptavidin binders from a DNA-encoded chemical library. *Bioconjug. Chem.*.

[29] Thermo Fisher Scientific (2022). Immobilized TCEP disulfide reducing gel: user guide. ..

[30] Esmaeili S., Rajil N., Hemmer P., Scully M. (2021). Monitoring of the gold nanoparticles during preparation processes. *Frontiers in Optics + Laser Science*.

[31] Esmaeili S. (2022). *Frontiers in Optics + Laser Science 2022 (FIO, LS)*.

[32] Sebaugh J. (2011). Guidelines for accurate EC50/IC50 estimation. *Pharm. Stat.*.

