# Supplementary material for: Quantum-enhanced detection of viral cDNA via luminescence resonance energy transfer using upconversion and gold nanoparticles
Source: Nanophotonics. 2025 Mar 31;14(23):3965–76. doi: 10.1515/nanoph-2024-0663 (PMC12617699; doi:10.1515/nanoph-2024-0663)
Supplement: Supplementary file 1 — Supplementary Material Details [file j_nanoph-2024-0663_suppl_001.pdf]

Supporting Information

Quantum-Enhanced Detection of Viral ccDNA via Luminescence Resonance Energy Transfer Using Upconversion and Gold Nanoparticles

Shahriar Esmaeili[1, 2], Navid Rajil[1, 2], Ayla Hazrathosseini [1, 2], Benjamin W. Neuman[3], Masfer H. Alkahtani[4], Dipankar Sen[1, 2], Qiang Hu[5], Hung-Jen Wu [5], Zhenhuan Yi[1, 2], Robert W. Brick [1, 2], Alexei V. Sokolov[1, 2, 6], Philip R. Hemmer [1, 2, 6], and Marlan O. Scully [1, 2]

[1]Institute for Quantum Science and Engineering, Texas A&M University, College Station, TX 77843, USA
[2]Department of Physics and Astronomy, Texas A&M University, College Station, TX 77843, USA
[3]Department of Biology, Texas A&M University, College Station, TX 77843, USA
[4]King Abdulaziz City for Science and Technology (KACST), Riyadh 11442, Saudi Arabia
[5]Department of Chemical Engineering, Texas A&M University, College Station, TX 77843, USA
[6]Department of Electrical and Computer Engineering, Texas A&M University, College Station, TX 77843, USA

Introduction

This document provides supplementary materials for our study on the interaction between gold nanoparticles (AuNPs) and upconversion nanoparticles (UCNP). It covers the following sections: Experimental Section (including materials and equipment, synthesis of Oleic Acid-Capped $LiYF_4$ particles, surface–ligand exchange of UCNPs, and characterization techniques like TEM and DLS analysis). Additionally, it includes detailed calculations and discussions on the absorption cross-section, cDNA quenching efficiency, and binding affinity.

1 Experimental Section

1.1 Materials and equipment

1.1.1 Reagents

Oleic acid (technical grade, 90%; Sigma-Aldrich, cat. no. 364525), 1-Octadecene (technical grade, 90%; Sigma-Aldrich, cat. no. O806), Methanol (reagent grade), Absolute Ethanol 200 proof (reagent grade), Cyclohexane (reagent grade), Deionized (DI) water, $Yb(CH_3CO_2)_3{\cdot}4H_2O$ (99.9% trace metals basis; Sigma-Aldrich, cat. no. 326011), $Tm(CH_3CO_2)_3{\cdot}xH_2O$ (99.9% trace metals basis; Sigma-Aldrich, cat. no. 367702), $Y(CH_3CO_2)_3{\cdot}xH_2O$ (99.9% trace metals basis; Sigma-Aldrich, cat. no. 326046), $Er(CH_3CO_2)_3{\cdot}4H_2O$ (99.9% trace metals basis; Sigma-Aldrich, cat. no. 207234-04-6), Lithium acetate (99.95% trace metals basis, cat. no. 517992), NH_4F (ACS reagent, >98%; Sigma-Aldrich, cat. no. 216011), Dialysis Tubing, 3.5K MWCO, 35 mm (cat. no.: 88244), Diethylene glycol (DEG 99%, Sigma), 2,2'-[ethylenebis(oxy)] bisacetic acid (cat. no. 23243-68-7). The chemical materials were used as is without any additional purification steps.

The oligonucleotide 5'- GAC CTA CAC AGG TGC CAT CAA ATT GGA TCA CAA AGA TCC AAA T-3' (as original target cDNA), 3 types of mismatch oligonucleotides including 5' - GAC CTA CAC AGG TGC CAT CAA ATT GAG AAT ACC GAT ACC AAA T- 3' (as target cDNA-mmP1: primer 2 but not primer 1 to bind, 12 mismatch bases), 5' - GAC CGC ACA CTC GTG AAT CAA ATT GGA TCA CAA AGA TCC AAA T- 3' (as target cDNA-mmP2: primer 1 but not primer 2 to bind, 12 mismatch bases), 5' - GAC CGC ACA CTC GTG AAT CAA ATT GAG AAT ACC GAT ACC AAA T - 3 (target cDNA-mmP1p2: neither primer to bind, 24

mismatch bases), 5'- /5ThioMC6-D/ATT TGG ATC TTT GTC ATC CAA T- 3' (as primer 1: 100-thiol-modified oligonucleotide), plus 5'- TTG ATG GCA CCT GTG TAG GTC /3AmMO/- 3' (primer 2: amino-101 modified oligonucleotide were synthesized by Integrated cDNA Technologies Inc. Spherical gold nanoparticles-5nm (product num: A11-5-CIT-DIH-1) was obtained from Nanopartz. Tris(2-carboxyethyl)phosphine hydrochloride (TCEP) was also purchased from Thermofisher Inc.

1.1.2 Equipment

Schlenk line, Magnetic stirrer, Heating mantle with temperature controller, Argon (99.9%) Three-neck 100-ml flask, Centrifuge, Transmission electron microscope (TEM, JEOL 1200), and carbon-coated copper TEM grids, Ultracentrifuge Beckman Coulter Optima MAX-XP. Polypropylene Tube (Product No:357448) and adapters (part number: 357448) for tubes in Beckman coulter rotors for use in ultracentrifuge rotor (TLA-110) were obtained from Beckman Coulter.

1.2 Synthesis of Oleic Acid-Capped $LiYF_4$:Yb^{3+}(18%), Er^{3+}(1.5%), Tm^{3+}(0.5%) core particles

In a 250 mL three-neck flask at room temperature, we combined 25 mL of oleic acid and 25 mL of 1-octadecene. This mixture was then degassed at 100 °C under vacuum conditions (approximately 0.8 torr) using a Schlenk-line, continuing until no more gases were released. Under an argon atmosphere, we added a combination of lithium acetate (10 mmol) and various rare earth acetates (5 mmol in total) to the degassed solvent. This included 4 mmol of $Y(CH_3CO_2)_3{\cdot}xH_2O$, 0.9 mmol of $Yb(CH_3CO_2)_3{\cdot}4H_2O$, 0.075 mmol of $Er(CH_3CO_2)_3{\cdot}4H_2O$, and 0.025 mmol of $Tm(CH_3CO_2)_3{\cdot}H_2O$.

The solution, continuously stirred, was then heated again to 100 °C under vacuum until the release of acetic acid ceased. Subsequently, the solution was heated to 300°C in an argon atmosphere for 10 minutes before being allowed to cool down to 100°C. A subsequent degassing step at 100 °C for an hour was performed to remove acetic acid generated during the heating phase. After purging the reactor with argon gas, we added 25 mmol of dry NH4F at 100 °C. The reactor was then subjected to three cycles of brief vacuum (2-3 mins) and argon refills to eliminate any air. The mixture was stirred at 300°C for 2 hours under argon, and upon cooling to room temperature, was centrifuged to yield a yellowish solution. The resultant nanoparticles were washed thrice with a 1:1 (v/v) mixture of cyclohexane and ethanol (4 mL each). Finally, the nanoparticles (20 mg/ml) were resuspended in 10 mL of fresh cyclohexane and stored at approximately 4 °C [Alkahtani et al., 2021, Carl et al., 2021].

1.2.1 Surface–ligand exchange of UCNPs by EBAA (2, 2'-[ethylenebis(oxy)] bisacetic acid)

A solution containing 500 mg of EBAA in 8 ml of diethylene glycol was first heated to 110 °C under an argon atmosphere, with continuous vigorous stirring. Subsequently, 1 ml of $LiYF_4$(18%), Er(1.5%), Tm(0.5%) core particles, equivalent to 20 mg and dissolved in cyclohexane, was introduced into the mixture. Upon the addition of 20 mg of UCNPs in a chloroform solution, the initially hot solution promptly turned cloudy. This mixture was then kept at 240 °C for about 1.5 hours until it transformed into a clear solution. Post-heating, the solution underwent dialysis in diethylene glycol (DEG) for 20 hours, aiming to eliminate excess ligands and solvents. Finally, the hydrophilic UCNPs were isolated by centrifugation, thoroughly washed with pure water, and redispersed in deionized water for further analysis. Figure S1 illustrates the synthesis of these hydrophilic upconversion nanoparticles (UCNPs) via the ligand exchange process using Enhanced Branched Amphiphilic Acid (EBAA) [Esmaeili et al., 2023].

1.2.2 Characterization: TEM and DLS Analysis

Transmission electron microscopy (TEM, using a JEOL 1200 instrument) was employed to analyze the surface characteristics of our samples. Additionally, the size of both oleate-coated and EBAA-capped upconversion nanoparticles (UCNPs) was determined using dynamic light scattering (DLS). As depicted in Figure (S2), TEM images showcase the differences between the original and EBAA-capped UCNPs. The original, unmodified

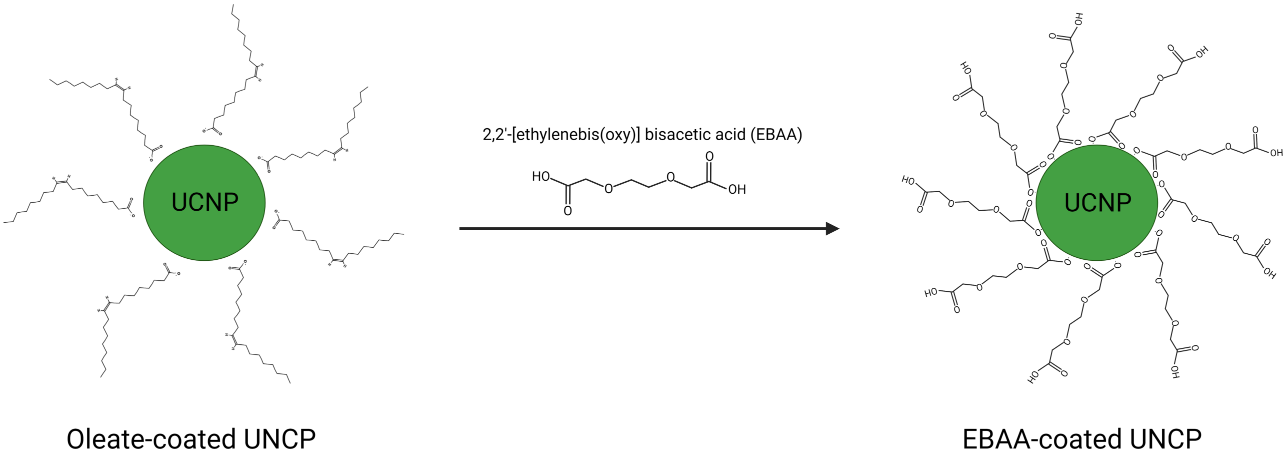

Figure S1. A schematic representation of the EBAA method, illustrating the ligand exchange process with EBAA, and resulting hydrophilic UCNP [Esmaeili et al., 2023].

UCNPs appear spherical and nearly monodisperse, with an average diameter around 12 nm, as seen in Fig. S2 (a). In contrast, the EBAA-capped UCNPs retain a similar size and shape to their unmodified versions, which is evident in Fig. S2 (b).

To complement the TEM analysis, dynamic light scattering (DLS) was used to examine the size distribution of the nanoparticles. The DLS size distribution of the original oleate-coated nanoparticles ($LiYF_4$(18%), Er (1.5%), Tm (0.5%)) dispersed in cyclohexane (Fig. S3 (a)) and the EBAA-capped UCNPs in water (Fig. S3 (b)) demonstrated the uniformity and monodispersity of the particles. Moreover, the DLS results indicated that the size distribution was slightly smaller than that suggested by the TEM images due to the effect of the surrounding medium and differences in measurement techniques. The successful surface modification suggested by the DLS analysis also contributed to the colloidal stability and compatibility of the nanoparticles [Classen et al., 2022, Sen et al., 2021, 2022].

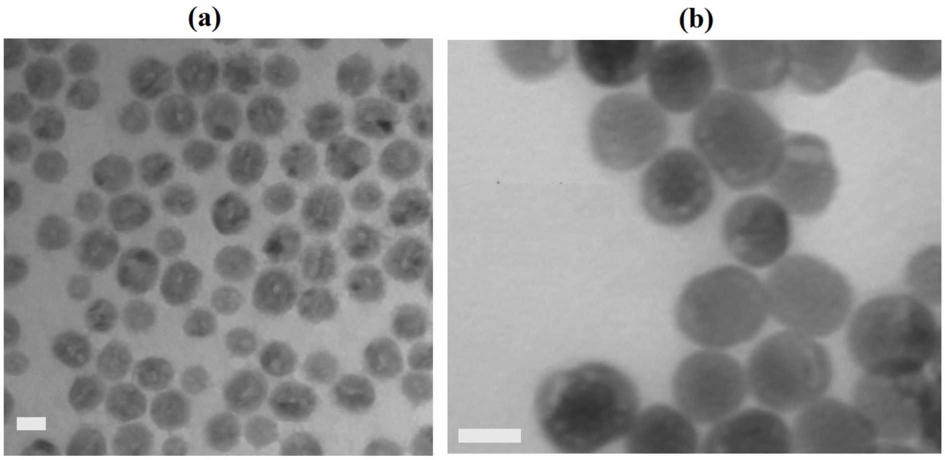

Figure S2. TEM images of $LiYF_4$(18%), Er(1.5%), Tm(0.5%) UCNPs captured before (a) and after (b) EBAA ligand exchange using a JEOL 1200 TEM. The magnification levels refer to zoom factors of 100,000x (a) and 200,000x (b). Scale bars of 11 are included in images (a) and (b), respectively, to provide a clear size reference.

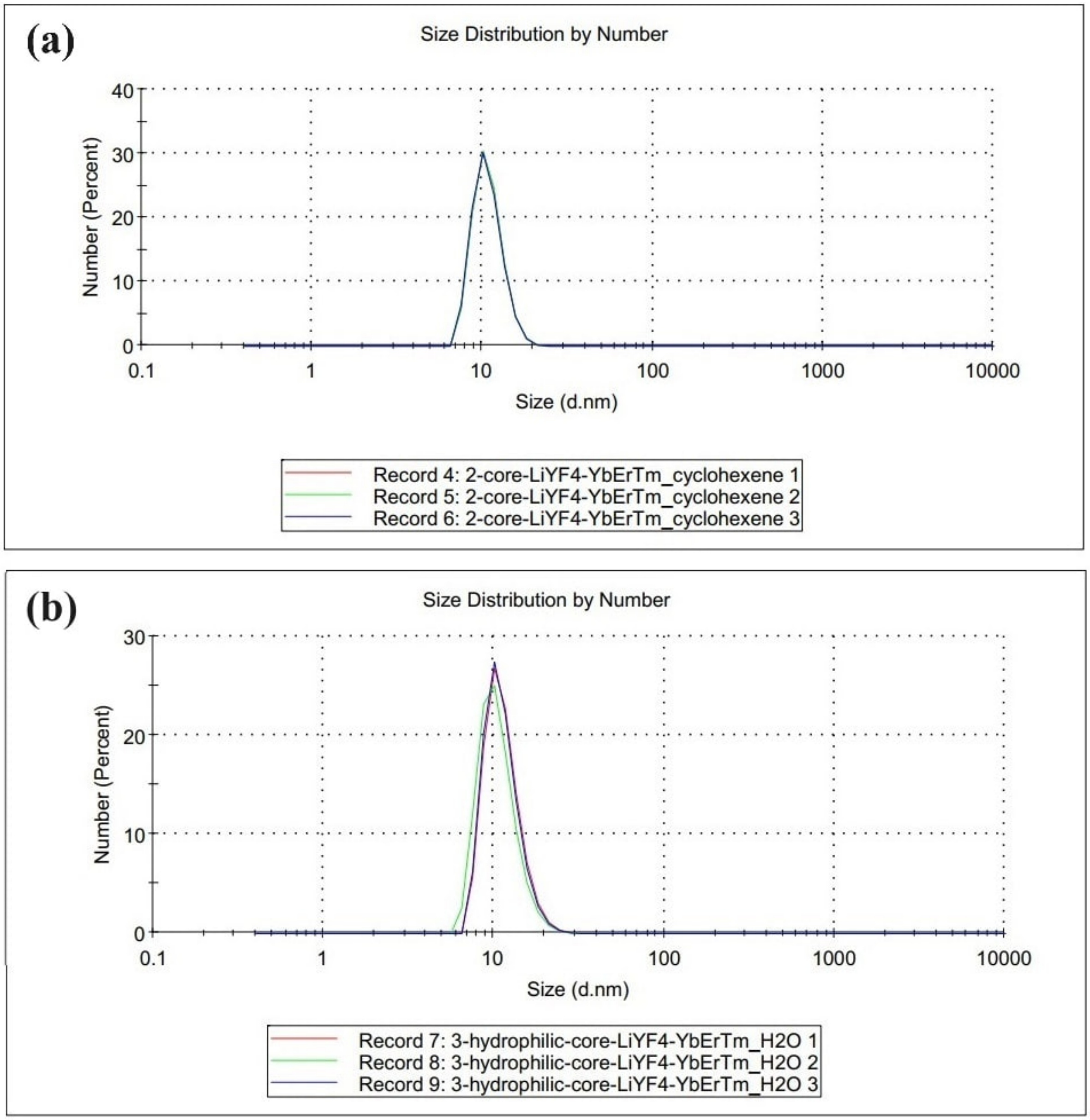

Figure S3. The size distribution of the (a) original oleate-coated nanoparticles ($LiYF_4$(18%), Er (1.5%), Tm (0.5%)) dispersed in cyclohexane was measured (size: 10.86 nm) as well as that of the (b) EBAA-capped UCNPs dispersed in DIH2O (size: 11.39 nm). [Esmaeili et al., 2023].

It is estimated that the density of the $LiYF_4$(18%), Er(1.5%), Tm(0.5%) particles with an 11 nm diameter is approximately $4.2 \times 10^{-18} mg/nm^3$. Based on this, it can be calculated that 1.3 mg of such particles contain approximately 4.67×10^{14} particles.

Figure S4 shows the TEM image of the positive control sample. As can be seen, a single UCNP is shown with several smaller AuNPs surrounding it. This image shows that the distance between the UCNP and AuNPs is small enough to be within the range of FRET coupling (reported to be below 1-10 nm Stryer and Haugland [1967], Lakowicz [2006]).

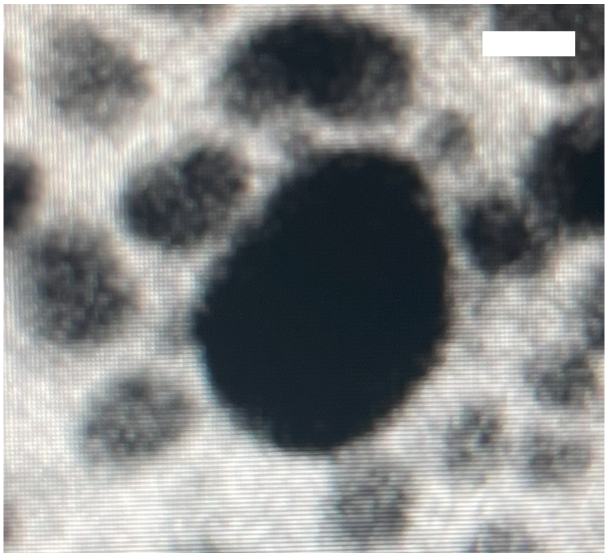

Figure S4. TEM images of positive control test, showing the larger UCNP attached to several smaller AuNPs (scale bar is 6 nm).

1.3 Zeta Potential Measurements

Zeta potential measurements were performed to evaluate the surface charge and stability of the nanoparticles under DI water conditions. The results are summarized below (Figure S5):

- **UCNPs:** Zeta potential: 6.48 mV (Figure S5a).
- **UCNP-primer conjugates:** Zeta potential: -24.1 mV (Figure S5b).
- **AuNPs:** Zeta potential: -31.2 mV (Figure S5c).
- **AuNP-primer conjugates:** Zeta potential: -38.4 mV (Figure S5d).

We performed the zeta potential measurements primarily to verify that the surface charge changes after coating the nanoparticles with primers, which serves as an indicator of successful conjugation (Figure S5). Our aim was not to obtain the absolute zeta potential values but to observe the difference before and after conjugation. Additionally, the dose-response curve derived from our experimental data confirms that the system functions as expected, supporting the robustness and effectiveness of our assay.

These measurements are consistent with those reported in the reference study by Tsang et al. [2016], affirming the stability and functionality of our nanoparticles under DI water conditions. Future studies will explore the performance of these nanoparticles in PBS.

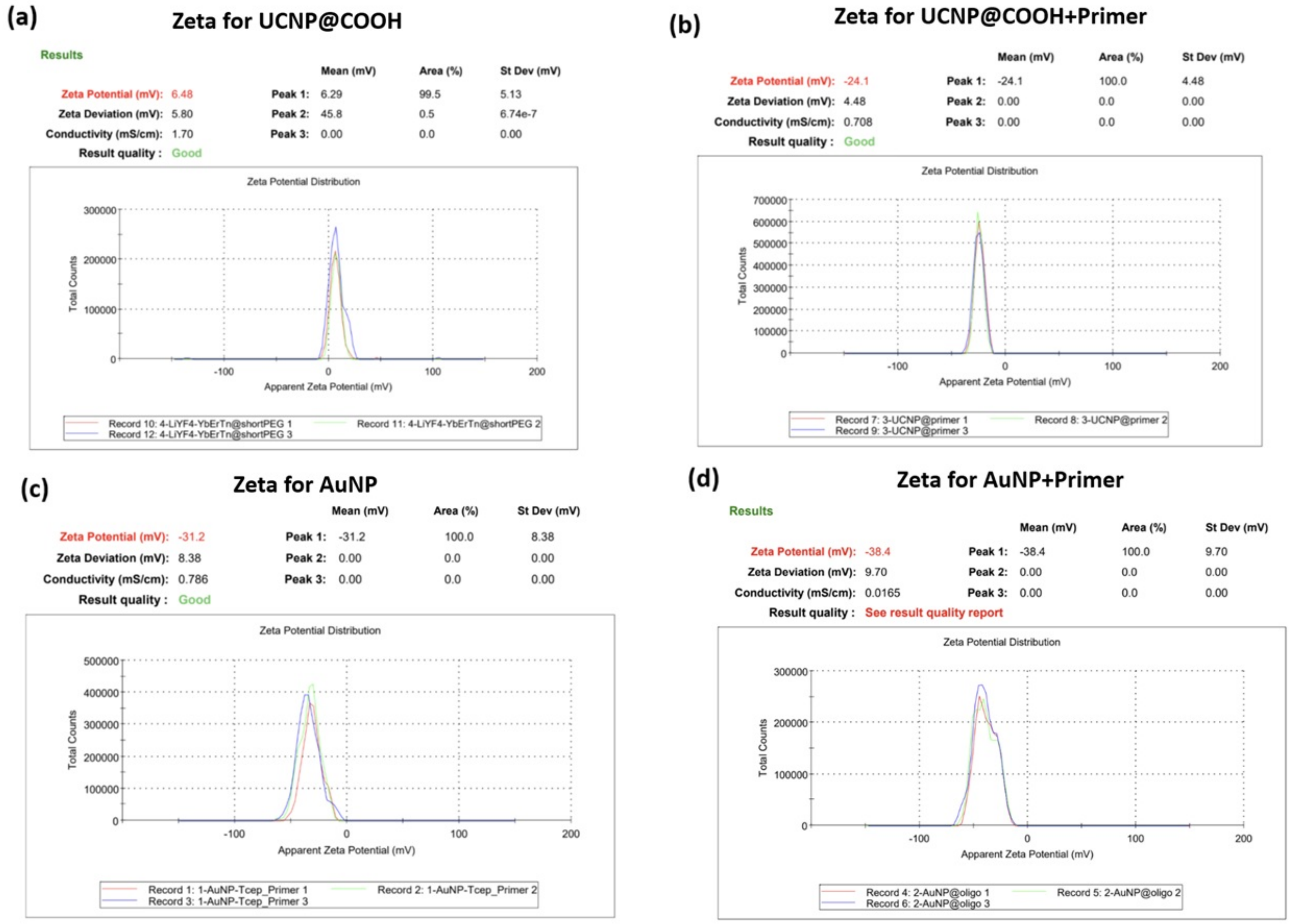

Figure S5. Zeta potential measurements: (a) Carboxylated UCNPs (6.47 mV); (b) UCNP-primer conjugates (-24.1 mV); (c) AuNPs (-31.2 mV); (d) AuNP-primer conjugates (-38.4 mV).

2 Ratio of Absorption Cross Section of AuNPs and Area of UCNP

An alternative estimation approach involves dividing the surface area of the UCNP by the absorption cross section of the AuNPs. As detailed in the study by Myroshnychenko et al. [2008] the absorption cross section for a gold nanoparticle with a diameter of 4.9 nm is approximately $5.0\,\mathrm{nm}^2$. For our UCNP, with a diameter of 15 nm, the surface area is calculated as $4\pi r^2 = 707\,\mathrm{nm}^2$. This results in a surface area that is 141 times greater than the absorption cross-section of the AuNPs.

3 Quenching Efficiency Analysis for Target and Mismatch cDNA Samples

3.1 Expected Quenching Efficiency for Target vs. Mismatched cDNA Samples

The quenching phenomenon originates from the binding interaction between AuNPs and UCNPs, mediated by the affinity of the cDNA targeted for detection and its concentration. The Langmuir equation provides a theoretical framework to describe this binding interaction:

$$\theta = \frac{[\text{cDNA.Primer}]}{[\text{Primer}]_{\text{total}}} \approx \frac{[\text{cDNA}]_{\text{total}}}{K_d + [\text{cDNA}]_{\text{total}}} \quad (1)$$

where [cDNA.Primer] represents the concentration of cDNA bound to both primers, $[\text{Primer}]_{\text{total}}$ denotes the total primer concentration (including UCNPs and AuNPs), and $[\text{cDNA}]_{\text{total}}$ is the total concentration of cDNA (target, mmP1, mmP2, or mmP1p2).

The affinity of a cDNA sequence for its primers depends on its degree of complementarity. The fully matched target cDNA exhibits the highest affinity, leading to maximum quenching. Single-mismatch sequences (mmP1 or mmP2) display reduced affinity and, consequently, lower quenching efficiency. The lowest affinity occurs with the double-mismatch sequence (mmP1P2), which leads to minimal quenching, approaching the control case where no cDNA is present. These trends are consistent with expected DNA hybridization physics and quenching behavior.

3.2 Qualitative Binding Affinity Trends

Although theoretical calculations can be used to estimate relative binding trends, precise experimental determination of dissociation constants (K_d) would be required to quantify these interactions accurately. In our study, we focus on qualitative trends rather than absolute values. The observed quenching efficiency follows the expected ranking:

$$\text{Target cDNA} > \text{cDNA-mmP1} > \text{cDNA-mmP2} > \text{cDNA-mmP1P2} \quad (2)$$

This ordering aligns with the principles of DNA hybridization, where greater sequence complementarity leads to stronger binding and increased quenching. However, it is important to note that mismatched sequences can still exhibit measurable binding activity, particularly at high cDNA concentrations, which is why quenching is still observed in cases with mismatches.

3.3 Role of Concentration in Quenching Efficiency

The observed quenching trends are also influenced by the concentration of cDNA in the assay. Even sequences with lower binding affinity (such as cDNA-mmP1P2) can still exhibit detectable quenching at high concentrations. This behavior is consistent with the Langmuir equation, which suggests that binding efficiency is dependent not only on affinity but also on the relative availability of cDNA molecules.

The Langmuir model predicts:

$$\theta = \frac{[\text{cDNA}]_{\text{total}}}{K_d + [\text{cDNA}]_{\text{total}}} \quad (3)$$

Given that the cDNA concentration in our experiment was significantly higher than the expected dissociation constants for mismatched sequences, measurable binding was still observed even in the case of cDNA-mmP1P2. This explains why quenching was not entirely suppressed, and some signal reduction was still detected at 550 nm.

References

M. Alkahtani, N. Alsofyani, A. Alfahd, A. A. Almuqhim, F. A. Almughem, A. A. Alshehri, H. Qasem, and P. R. Hemmer. Engineering red-enhanced and biocompatible upconversion nanoparticles. *Nanomaterials*, 11(2):284, 2021.

F. Carl, L. Birk, B. Grauel, M. Pons, C. Würth, U. Resch-Genger, and M. Haase. Liyf 4: Yb/liyf 4 and liyf 4: Yb, er/liyf 4 core/shell nanocrystals with luminescence decay times similar to ylf laser crystals and the upconversion quantum yield of the yb, er doped nanocrystals. *Nano Research*, 14:797–806, 2021.

A. Classen, D. Sen, L. Grüner-Nielsen, H. C. Gibbs, S. Esmaeili, P. Hemmer, A. Baltuska, A. V. Sokolov, R. A. Leitgeb, A. Fernández, et al. Modeling the image formation process in fourier domain optical coherence microscopy for a bessel-like lp02 mode from a higher order mode fiber. In *CLEO: QELS_Fundamental Science*, pages JW3A–3. Optica Publishing Group, 2022.

S. Esmaeili, N. Rajil, A. Hazrathosseini, B. W. Neuman, M. H. Alkahtani, Y. A. Alzahrani, Z. Yi, R. W. Brick, A. V. Sokolov, P. R. Hemmer, et al. Innovations in surface modification techniques: Advancing hydrophilic\textit {LiYF _{4}: Yb, Er, Tm} upconversion nanoparticles and their applications. *arXiv preprint arXiv:2312.07303*, 2023.

J. R. Lakowicz. *Principles of fluorescence spectroscopy*. Springer, 2006.

V. Myroshnychenko, J. Rodríguez-Fernández, I. Pastoriza-Santos, A. M. Funston, C. Novo, P. Mulvaney, L. M. Liz-Marzán, and F. J. G. De Abajo. Modelling the optical response of gold nanoparticles. *Chemical Society Reviews*, 37(9):1792–1805, 2008.

D. Sen, A. Classen, A. Fernández, L. Grüner-Nielsen, H. C. Gibbs, S. Esmaeili, P. Hemmer, A. Baltuska, A. V. Sokolov, R. A. Leitgeb, et al. Extended focal depth fourier domain optical coherence microscopy with a bessel-beam–lp 02 mode–from a higher order mode fiber. *Biomedical Optics Express*, 12(12):7327–7337, 2021.

D. Sen, A. Classen, L. Grüner-Nielsen, H. C. Gibbs, S. Esmaeili, P. Hemmer, A. Baltuska, A. V. Sokolov, R. A. Leitgeb, A. Fernández, et al. Implementation of bessel-like lp 02 mode from higher order mode (hom) fiber to extend the depth of focus in fourier domain optical coherence microscopy (fd-ocm). In *2022 Conference on Lasers and Electro-Optics (CLEO)*, pages 1–2. IEEE, 2022.

L. Stryer and R. P. Haugland. Energy transfer: a spectroscopic ruler. *Proceedings of the National Academy of Sciences*, 58(2):719–726, 1967.

M.-K. Tsang, W. Ye, G. Wang, J. Li, M. Yang, and J. Hao. Ultrasensitive detection of ebola virus oligonucleotide based on upconversion nanoprobe/nanoporous membrane system. *Acs Nano*, 10(1):598–605, 2016.
